# Volume-guarantee vs. pressure-limited ventilation in evolving bronchopulmonary dysplasia

**DOI:** 10.3389/fped.2022.952376

**Published:** 2022-12-23

**Authors:** Milenka Cuevas Guaman, Joseph Hagan, Dajana Sabic, Davlyn M. Tillman, Caraciolo J. Fernandes

**Affiliations:** Department of Pediatrics, Division of Neonatology, Texas Children’s Hospital, Baylor College of Medicine, Houston, TX, United States

**Keywords:** BPD (bronchopulmonary dysplasia), ventilaion, volume-guaranteed ventilation, pressure-limited ventilation, premature

## Abstract

**Introduction:**

Extremely premature infants are at high risk for developing bronchopulmonary dysplasia (BPD). While noninvasive support is preferred, they may require ventilator support. Although volume-targeted ventilation (VTV) has been shown to be beneficial in preventing BPD, no data exists to guide ventilator management of infants with evolving BPD. Thus, clinicians employ a host of ventilator strategies, traditionally time-cycled pressure-limited ventilation (PLV) and more recently volume-guarantee ventilation (VGV) (a form of VTV). In this study, we sought to test the hypothesis that use of VGV in evolving BPD is associated with improved clinical and pulmonary outcomes when compared with PLV.

**Design:**

Single-center, retrospective cohort review of premature infants born less than 28 weeks inborn to a Level 4 NICU from January 2015 to December 2020. Data abstracted included demographics, maternal and birth data, and ventilator data until death or discharge. Exposure to either VGV or PLV was also examined, including ventilator “dose” (number of time points from DOL 14, 21 and 28 the patient was on that particular ventilator) during the period of evolving BPD.

**Results:**

Of a total of 471 patients with ventilation data available on DOL 14, 268 were not ventilated and 203 were ventilated. PLV at DOL 21 and 28 was associated with significantly higher risk of BPD and the composite outcome of BPD or death before 36 weeks compared to VGV. Both increasing VGV and PLV doses were significantly associated with higher odds of BPD and the composite outcome. For each additional time point of VGV and PLV exposure, the predicted length of stay (LOS) increased by 15.3 days (*p* < 0.001) and 28.8 days (*p* < 0.001), respectively.

**Discussion:**

Our study demonstrates the association of use of VGV at DOL 21 and 28 with decreased risk of BPD compared to use of PLV. Prospective trials are needed to further delineate the most effective ventilatory modality for this population with “evolving” BPD.

## Introduction

Extremely premature infants (< 28 weeks gestation at birth, EPIs) are at high risk for developing a chronic lung disease termed bronchopulmonary dysplasia (BPD) (1–4). Despite numerous technological advances, the incidence, clinical course, and management of infants with BPD remain poorly defined and extremely variable (5–8). To standardize care for this high-risk group of infants, in 2001, the National Institute of Child Health and Human Development (NICHD) workshop proposed a definition of BPD defining BPD by the treatment received at 28 days of life (DOL) and 36 weeks postmenstrual age (PMA) (2, 9–11). This definition has been validated in multiple studies and is useful in predicting long-term pulmonary outcomes (12). Recently Jensen et al. have updated this definition (8). While standardizing terminology and definitions used in the management of patients with BPD is important, it is only the first step in improving care (10, 13, 14). A necessary next step to improving clinical outcomes and preventing BPD is understanding the pathophysiological mechanisms that cause BPD in the high-risk EPI population, as not all EPIs develop BPD. This understanding will lead us to treat EPIs differently to address their underlying susceptibilities, which could decrease the incidence of the disease. Management strategies to address such underlying susceptibilities before an infant's birth include treating maternal uterine infections, antenatal steroids, and tocolytics (15–18). Regarding ventilatory strategies to prevent BPD, only using volume-targeted ventilation (VTV) when invasive respiratory support is unavoidable has been suggested (19, 20).

Ventilator management of the EPI has been described extensively, with the acknowledged best strategy being avoiding invasive ventilation altogether (21–23). However, in a subset of EPIs, invasive ventilation is unavoidable and/or attempts at extubation fail, and these infants subsequently may develop BPD (5, 24, 25). Currently, there are no optimal methods to screen for or guide the management of this vulnerable cohort, especially with regards to ventilator management (26, 27). Not surprisingly, clinicians employ a host of ventilator strategies, traditionally time-cycled pressure-limited ventilation (PLV) and VTV (19, 28). When used in the immediate postnatal period, VTV has been shown to decrease the incidence of BPD in EPIs (20, 29, 30), however, the utility of VTV in EPIs with evolving and/or established BPD has not yet been investigated. To address this knowledge gap, in this study, we sought to investigate the clinical and pulmonary outcomes of EPIs with evolving BPD treated with either VTV or PLV. Specifically, in this retrospective study, we tested the hypothesis that use of volume-guarantee ventilation (VGV - a form of VTV) would result in improved clinical and pulmonary outcomes when compared with use of PLV in a high-risk cohort of EPIs.

## Materials and methods

Data Extraction and Patient Selection: We identified all infants born at less than 28 weeks gestation who were admitted to the Pavilion for Women at Texas Children's Hospital from January 2015 to December 2020 using the institutional database that is submitted to the Vermont Oxford Network (VON). All patient information in this registry is de-identified. The definition we utilize for BPD is the one described in the NICHD workshop in 2001 at 36 weeks PMA. At our institution, if EPIs need ventilation at birth, our primary mode of ventilation is Assist-Control/Volume-Guarantee (AC-VG) with an attempt to extubate babies to nasal continuous positive airway pressure (NCPAP) in the first week of life or as soon as possible. Infants who fail to be extubated stay on AC-VG with continued attempts to extubate them. If they continue to fail, often somewhere in the 3rd or 4th week of life, alternative modes of ventilatory support may be considered, depending on the biases of the clinician on service and their management of the infant at the time. The usual rotation of an attending in the NICU is between 2 and 4 weeks but, to minimize the variability in care delivered, these infants are usually cared for in a small baby unit with a subset of neonatologists and other dedicated personnel. Ventilator management and non-invasive respiratory support provided to this population followed evidence-informed consensus-based guidelines that are reviewed annually, however the use of postnatal steroids for ventilator-dependent EPIs is variable and practitioner-dependent.

We subsequently conducted a chart review *via* an EPIC data extract to retrieve clinically relevant information, including demographic and respiratory support/ventilator data, using a standardized clinical data collection form for each patient weekly between birth and 36 weeks PMA and/or discharge date or death, if earlier. All data were collated in a de-identified datasheet for analyses.

Inclusion Criteria: Infants with gestational age (GA) less than 28 weeks at birth with mechanical ventilation data available at DOL 14, from January 2015 to December 2020.

Exclusion Criteria: Infants with GA 28 weeks or greater at birth, infants with major congenital or genetic anomalies, including congenital diaphragmatic hernia, congenital heart disease, myelomeningocele, or trisomies.

Data Analyses: The analytic cohort was restricted to patients with ventilator status data (PLV, VGV, Other) available for DOL 14. These patients' ventilator status at DOL 21 and 28 was also ascertained when data was available. A “VGV dose” variable was created for each patient that was calculated as the number of VGV ventilations across the three time points (DOL 14, 21 and 28), so the VGV dose could range from a score of 0 to 3 across these three time points. A “PLV dose” variable was computed in the same way. To control for differences in severity of lung disease, we used a previously validated respiratory severity score (RSS) (31). In addition to the unadjusted analyses, logistic regression analysis was used to compare odds of BPD, death before 36 weeks PMA and the composite of these two outcomes for patients on PLV vs. VGV at DOL 14, 21 and 28, after controlling for time period (2015–2017 vs. 2018–2020) and RSS at the corresponding DOL. Similarly, logistic regression was used to examine the associations of PLV and VGV dose exposures both before and after controlling for RSS at DOL 14 and time period (2015–2017 vs. 2018–2020).

Quantitative variables were assessed for normality using the Shapiro-Wilk test. Variables that were not normally distributed were compared between groups using the Wilcoxon rank sum test while normally distributed variables were compared using the two-sample t-test. Categorical variables were compared using Fisher's exact test. Logistic regression was used to examine associations of ventilator dose with binary outcomes and linear regression was used for continuous outcomes. The Cochran-Armitage trend test was used to test for a temporal relationship with ventilator modes. SAS version 9.4 (SAS Institute Inc., Cary, North Carolina) was used for data analysis. A 5% significance level was used for all hypothesis tests.

## Results

**Figure 1:**
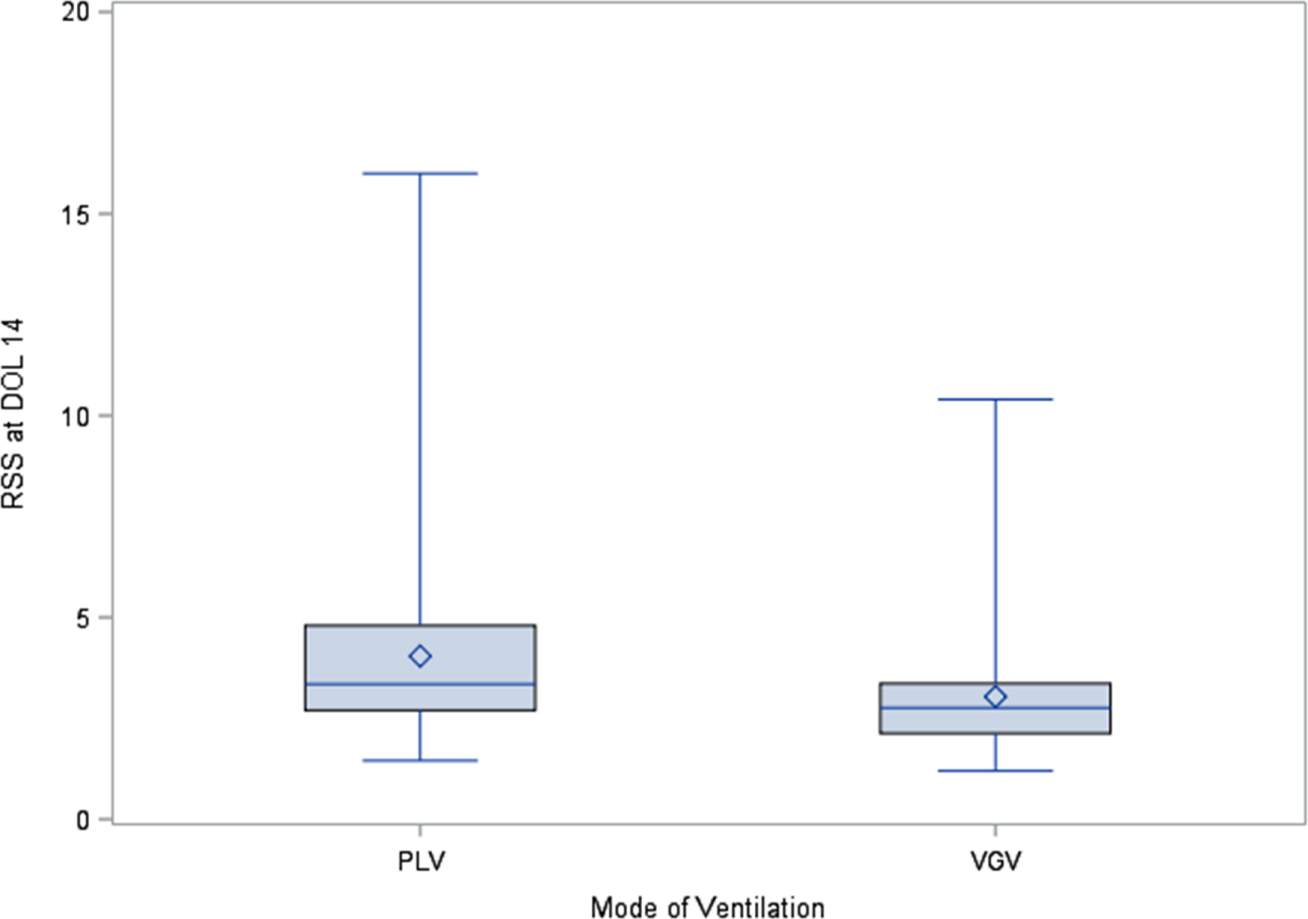
Boxplots of RSS at DOL 14 for patients on PLV and VGV.

There were 471 patients with mechanical ventilation data available at DOL 14. Of the 471 patients analyzed, 203 (43.1%) were on mechanical ventilation at DOL 14 and 268 (56.9%) were not. Patients who were on mechanical ventilation at DOL 14 had a significantly lower birthweight, lower GA, lower Apgar scores at 1 min and 5 min, as well as a significantly longer length of stay (LOS) (Table 1). RSS at DOL 14 were significantly (*p* < 0.01) higher for the 67 patients on PLV (median = 3.3, IQR: 2.7–4.8) compared to the 113 patients on VGV (median = 2.8, IQR: 2.1–3.4, Figure 1).

**Table 1:** Comparison of patients who were vs. were not on a ventilator at day of life 14.

Characteristic	Not on Ventilator at 14 Days of Life (*n* = 268)	On Ventilator (VGV, PLV or Other) at Day of Life 14 (*n* = 203)	*p*-value
Gender^a^			0.10
Female	145 (54.1)	94 (46.3)	
Male	123 (45.9)	109 (53.7)	
Maternal race^a^			0.40
Black	89 (33.2)	66 (32.5)	
White	161 (60.1)	118 (58.1)	
Asian	18 (6.7)	17 (8.4)	
American Indian	0 (0)	2 (1.0)	
Hispanic ethnicity^a^	90 (33.6)	59 (29.1)	0.32
Birthweight (g)^b^	890.0 (752.5, 1007.5)	675.0 (585.0, 794.0)	<0.01
GA at birth (weeks)^b^	26.7 (25.7, 27.3)	24.9 (24.0, 25.9)	<0.01
Apgar score at 1 min^b^	4 (2, 6)	3 (1, 5)	<0.01
Apgar score at 5 min^b^	7 (6, 8)	7 (5, 8)	<0.01
Antenatal steroids^a^	255 (95.2)	190 (93.6)	0.54
Chorioamnionitis^a^	32 (11.9)	24 (11.8)	1.00
Maternal hypertension^a^	84 (31.3)	72 (35.5)	0.37
Multiple gestation^a^	71 (26.5)	68 (33.5)	0.10
LOS (days)	93.0 (78.0, 113.0)	143.5 (119.0, 208.0)	<0.01

^a^ Frequency (%), Fisher's exact test *p*-value.

^b^ Median (inter-quartile range), Wilcoxon rank sum test *p*-value.

When comparing patients mechanically ventilated that were on VGV vs. PLV at DOL 14, there was no significant difference between BPD and the composite outcome of BPD or death before 36 weeks. Notably, PLV at DOL 21 was associated with significantly higher risk of BPD and the composite outcome of BPD or death before 36 weeks compared to VGV. Similarly, at DOL 28, PLV was associated with significantly higher risk of BPD and the composite BPD or death before 36 weeks outcome compared to VGV. After controlling for time period (2015–2017 vs. 2018–2020) and RSS at the corresponding DOL, odds of BPD and the composite outcome remained higher at DOL 21 and 28 for patients on PLV (Table 2). Examining the 180 patients on VGV or PLV at DOL 14, there was a significant (*p* < 0.01) increasing trend in the relative proportion of patients who were on VGV over the 6-year study period (Table 3).

**Table 2:** Risk of outcomes for VGV vs. PLV.

	On PLV	On VGV	*p*-value	Adjusted *p*-value^a^
At 14 days of life	(*n* = 67)	(*n* = 113)		
BPD	48 (71.6%)	85 (75.2%)	0.603	0.18
Death before 36 weeks PMA	9 (13.4%)	8 (7.1%)	0.191	0.13
Death before 36 weeks PMA or BPD composite	57 (85.1%)	93 (82.3%)	0.684	0.64
At 21 days of life	(*n* = 101)	(*n* = 93)		
BPD	87 (86.1%)	62 (66.7%)	0.002	0.04
Death before 36 weeks PMA	6 (5.9%)	7 (7.5%)	0.777	1.00
Death before 36 weeks PMA or BPD composite	93 (92.1%)	69 (74.2%)	0.001	0.02
At 28 days of life	(*n* = 105)	(*n* = 75)		
BPD	92 (87.6%)	52 (69.3%)	0.004	0.04
Death before 36 weeks PMA	5 (4.8%)	5 (6.7%)	0.744	0.68
Death before 36 weeks PMA or BPD composite	97 (92.4%)	57 (76.0%)	0.003	0.02

^a^ Adjusted for time period (2015–2017 vs. 2018–2020) and RSS at corresponding DOL.

**Table 3:** Frequency (%) of patients on PLV and VGV by year among the 180 patients on PLV or VGV at DOL 14.

Year	On PLV	On VGV
2015	23 (74.2%)	8 (25.8%)
2016	26 (55.3%)	21 (44.7%)
2017	4 (15.4%)	22 (84.6%)
2018	2 (7.1%)	26 (92.9%)
2019	3 (15.0%)	17 (85.0%)
2020	9 (32.1%)	19 (67.9%)
Total	67 (37.2%)	113 (62.8%)

Among the 471 patients with ventilator status data available at DOL 14, both increasing VGV dose and increasing PLV dose were associated with significantly increased odds of BPD, death before 36 weeks PMA, and the composite outcome of BPD or death before 36 weeks PMA (Table 4). After controlling for time period (2015–2017 vs. 2018–2020) and RSS at DOL 14, VGV dose was no longer significantly associated with BPD or the composite outcome, but increasing PLV dose was still associated with increased odds of these two outcomes. Neither VGV nor PLV dose was significantly associated with death before 36 weeks PMA after controlling for time period (2015–2017 vs. 2018–2020) and RSS at DOL 14 (Table 4).

**Table 4:** Association with outcomes of each additional week of exposure to VGV and PLV between DOL 14 and 28.

Exposure and Outcome	Ventilator Dose Exposure Odds Ratio (95% CI)	*p*-value	Adjusted Odds Ratio (95% CI)^a^	Adjusted *p*-value^a^
**VGV exposure**				
BPD	1.66 (1.30–2.12)	<0.01	0.891 (0.647–1.228)	0.48
Death before 36 weeks PMA	2.03 (1.10–3.76)	0.02	0.936 (0.419–2.090)	0.87
BPD or Death before 36 weeks PMA	1.74 (1.38–2.20)	<0.01	0.887 (0.650–1.209)	0.45
**PLV exposure**				
BPD	3.41 (2.62–4.43)	<0.01	1.911 (1.392–2.625)	<0.01
Death before 36 weeks PMA	1.93 (1.08–3.46)	0.03	1.665 (0.767–3.612)	0.20
BPD or Death before 36 weeks PMA	3.44 (2.66–4.44)	<0.01	2.008 (1.463–2.755)	<0.01

^a^ Adjusted for time period (2015–2017 vs. 2018–2020) and RSS at DOL 14.

**Figure 2:**
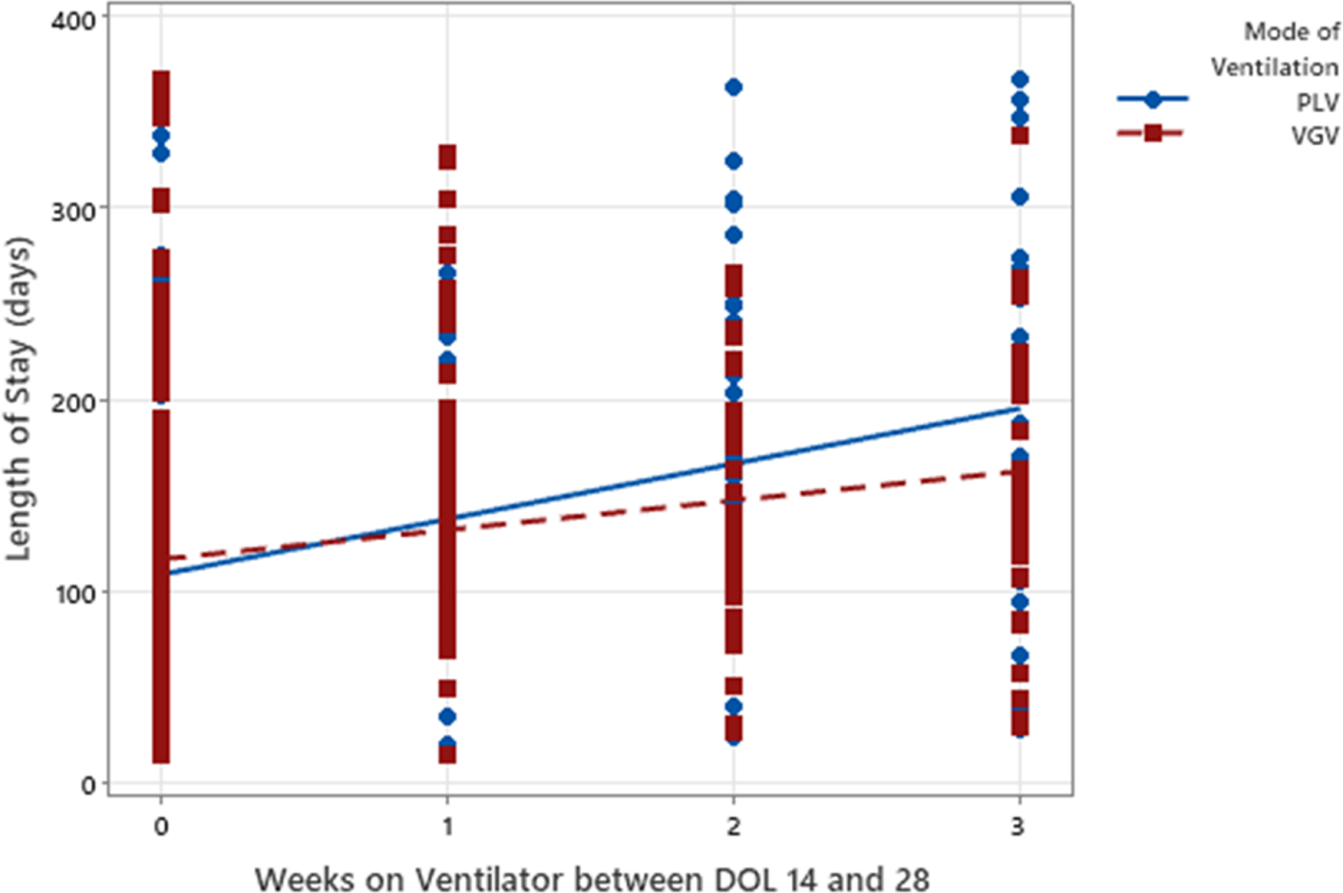
Scatterplot of weeks on VGV or PLV vs. LOS with linear regression line superimposed.

For every additional week of VGV, the predicted LOS increased by 15.3 days (*p* < 0.01, Figure 2). For every additional week of PLV, the predicted LOS increased by 28.8 days (*p* < 0.01, Figure 2).

## Discussion

This is a retrospective chart review of the medical records of EPIs admitted to a tertiary-care Level 4 NICU over a 6-year period who were ventilated during the first month of life. In this study, we found that EPIs ventilated at DOL14 were more likely to develop BPD, and at DOL 21 and 28, those treated with PLV had significantly higher incidence of BPD compared to those treated with VGV.

Recent trends in neonatal care show increased survival among infants born at lower GA (23–24 weeks), and underscores a need to focus on intervention strategies for the most immature infants (32, 33). Our findings highlight the fact that infants born at the lower extremes of birth weight and GA remain at increased risk for prolonged hospitalization and significant pulmonary morbidities. Due to this, there is a continued need to better understand the factors that might adversely impact outcomes.

As prior studies have focused on prevention of BPD and management strategies in established BPD, in our study, we chose to evaluate a unique time period in the hospitalization of EPIs, where the risk of mortality may have diminished but the risk of significant morbidity still remains (34, 35). Given that many infants of low birth weight and gestation may die before DOL 14, we chose to examine the 3rd and 4th week of life to focus on EPIs with “evolving BPD”. While the diagnosis of BPD has historically been assessed at 36 weeks PMA (8, 12, 36, 37), the optimal timing to transition management strategies from disease prevention to providing optimal long-term care of those with established BPD remains unknown. This is particularly true for the subset of infants with severe BPD (sBPD) as described in the literature (1, 38). As lung pathophysiology changes from that of respiratory distress syndrome to “evolving BPD”, ventilator management needs to change to match an infant's needs (5, 7, 39). Further research is however needed to determine what the optimal strategies are and when they should be implemented to minimize further lung injury and improve outcomes.

In our cohort, EPIs receiving mechanical ventilation at DOL 14, 21 and 28 had significantly higher probability of BPD, death before 36 weeks PMA, and composite outcome of BPD or death prior to 36 weeks. After controlling for RSS at DOL 14, increasing PLV dose, but not VGV dose, was still associated with significantly increased odds of BPD and the composite outcome. Neither VGV nor PLV dose was associated with death before 36 weeks PMA after controlling for RSS at DOL 14. This association implies that the pathophysiologic mechanisms underpinning the need for PLV persist in this subpopulation and/or PLV itself could incite lung injury that prolongs the need for ventilation. Recognizing this association is important because EPIs who require protracted mechanical ventilation are known to have significant increased morbidity and mortality over time, whether they remained on positive pressure ventilation at initial hospital discharge or not (40, 41). It is also important to note that more premature babies (23–25 wk GA) are more likely to be ventilated on DOL 14 than less premature babies (26–28 wk GA); this can affect BPD, BPD/death, or hospital LOS as a confounding variable. The likelihood of death or BPD in this cohort has been modeled using RSS at DOL 14, 21 and 28 (42). In light of this, attempts to ameliorate further lung injury are both warranted and should be attempted wherever possible.

While newborn premature infants ventilated at birth using VTV modes have been reported to have reduced rates of death, BPD, pneumothoraces, and duration of ventilation when compared with infants ventilated using PLV modes, the optimal ventilator strategy for infants with evolving or established BPD has not yet been determined (20, 29, 30). In our cohort, the number of babies on VGV on DOL 21 and DOL 28 were lower than on DOL14 whereas the number of babies on PLV was higher on DOL 21 and DOL 28 than on DOL14, although the relative proportion of infants ventilated with VGV increased over the 6-year period. One interpretation for this finding is that clinicians who did not find that VGV worked sufficiently well for their patients changed from VGV to PLV (5, 24, 38). In comparing patients mechanically ventilated on VGV vs. PLV, PLV at DOL 21 and 28 was associated with significantly higher risk of BPD and the composite BPD or death before 36 weeks compared to VGV. This is consistent with other reports of VTV reducing death or BPD (20). Infants requiring prolonged ventilation have a diverse airway and parenchymal and vascular phenotypes that may contribute to the need for PLV as a mode for effective ventilation. As such, as there are currently no standardized ventilator management guidelines for established BPD, the decision to use PLV or VGV is at the discretion of the clinician. In our cohort, we found a temporal shift over the 6-year period in practitioners' use of VGV over PLV from 26% in 2015 to 63% VGV by 2020 (Table 4). Additional studies are required to evaluate patient- and provider-specific clinical factors related to the use of VGV vs. PLV. Prospective trials are also needed to determine which invasive positive pressure ventilation (IPPV) or non-invasive support strategies are most effective in supporting infants with established BPD (6, 14, 43).

Although necessary for some patients, the use of PLV carries a theoretical risk of continuing volutrauma in lung units with greater compliance than others, as well as the risk of prolonging need for ventilator support. Indeed, while we found that increased duration of exposure to both VGV and PLV were associated with significantly increased odds of BPD, death before 36 weeks PMA, and the composite outcome, for each additional week of exposure, infants treated with PLV had almost twice the predicted increase in LOS compared to infants treated with VGV. While increased exposure to invasive ventilation is known to be associated with poor long-term outcomes (8, 43–45), whether and what part of this association is the result of ventilator-induced lung injury is yet to be determined (46–51).

We acknowledge some limitations in our study. As a single-center study, we lack data from other institutions and our patient demographics may be different compared to other NICUs, potentially limiting the generalizability of our observations. As a retrospective cohort study, our study also lacks randomization due to the observational nature. The initial analysis compared two groups: infants at DOL 14 who did not require ventilator support and infants who continued requiring mechanical ventilation, receiving either VGV, PLV or another ventilator strategy. Also due to the nature of our study and the hypothesis proposed, infants not requiring mechanical ventilation at DOL 14 were utilized as our control group. The VGV and PLV dose variables only quantify the number of time points among DOL 14, 21 and 28 that the patient was on that particular ventilation mode, which does not guarantee the patient was on that same type of ventilation mode for the entire week. In addition, it did not quantify the level of VGV and PLV support since the details of ventilator support such as mean airway pressure and achieved TV were not collected as well as not including other morbidities as PDA or sepsis. Furthermore, our results do not identify cause-effect and are reported as associations. Nevertheless, our study has some important strengths, including a large cohort of preterm patients with respiratory distress syndrome and protracted ventilation and data abstracted by a dedicated data team using standardized VON definitions. To our knowledge, our study is unique in examining the use of VGV vs. PLV in EPIs with “evolving BPD” and in demonstrating the association of use of VGV with decreased risk of BPD at DOL 21 and 28, compared to PLV.

Our findings suggest that the use of VGV rather than PLV for intubated EPIs during evolving BPD may lead to better clinical outcomes. We caution however, that our observed association of PLV with increased risk of adverse outcome in our cohort does not axiomatically imply a causal relationship. Prospective randomized trials are unquestionably needed to further delineate the most effective ventilatory modality for EPIs with “evolving” BPD.

## Data Availability

The raw data supporting the conclusions of this article will be made available by the authors, without undue reservation.
